# Tunable Subnanometer Gaps in Self-Assembled Monolayer Gold Nanoparticle Superlattices Enabling Strong Plasmonic Field Confinement

**DOI:** 10.1021/acsnano.3c03804

**Published:** 2023-06-24

**Authors:** Bin Lu, Karol Vegso, Simon Micky, Christian Ritz, Michal Bodik, Yuriy Myronovych Fedoryshyn, Peter Siffalovic, Andreas Stemmer

**Affiliations:** † Nanotechnology Group, ETH Zürich, Säumerstasse 4, CH-8803 Rüschlikon, Switzerland; ‡ Institute of Physics SAS, Dubravska cesta 9, 84511 Bratislava, Slovakia; § Institute of Electromagnetic Fields, ETH Zürich, Gloriastrasse 35, CH-8092 Zürich, Switzerland

**Keywords:** gold nanoparticle superlattices, subnanometer gap, ligand exchange, molecular junction, plasmonic coupling, SERS, optical metasurfaces

## Abstract

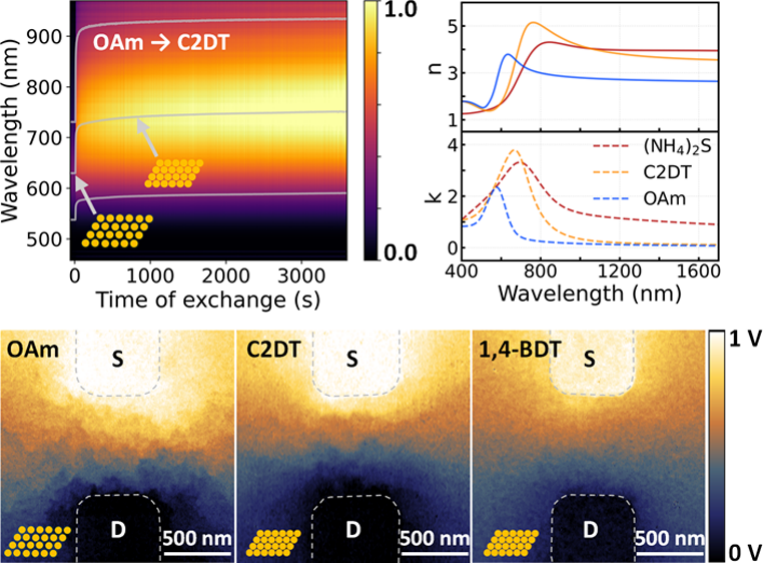

Nanoparticle superlattices produced with controllable interparticle gap distances down to the subnanometer range are of superior significance for applications in electronic and plasmonic devices as well as in optical metasurfaces. In this work, a method to fabricate large-area (∼1 cm^2^) gold nanoparticle (GNP) superlattices with a typical size of single domains at several micrometers and high-density nanogaps of tunable distances (from 2.3 to 0.1 nm) as well as variable constituents (from organothiols to inorganic S^2–^) is demonstrated. Our approach is based on the combination of interfacial nanoparticle self-assembly, subphase exchange, and free-floating ligand exchange. Electrical transport measurements on our GNP superlattices reveal variations in the nanogap conductance of more than 6 orders of magnitude. Meanwhile, nanoscopic modifications in the surface potential landscape of active GNP devices have been observed following engineered nanogaps. *In situ* optical reflectance measurements during free-floating ligand exchange show a gradual enhancement of plasmonic capacitive coupling with a diminishing average interparticle gap distance down to 0.1 nm, as continuously red-shifted localized surface plasmon resonances with increasing intensity have been observed. Optical metasurfaces consisting of such GNP superlattices exhibit tunable effective refractive index over a broad wavelength range. Maximal real part of the effective refractive index, *n*_max_, reaching 5.4 is obtained as a result of the extreme field confinement in the high-density subnanometer plasmonic gaps.

Individual metallic nanoparticles (NPs) can support localized surface plasmon resonances (LSPRs), which allow electromagnetic (EM) field confinement to subwavelength dimensions. Additionally, the plasmonic capacitive coupling of adjacent NPs can enhance such a field confinement. Consequently, local EM field enhancement by 2–5 orders of magnitude can be achieved in the nanogaps.^1,2^ Once it became possible to reduce the size of plasmonic nanogaps to (sub)nanometer scale, the strong light–matter interactions in nanogaps immensely boosted various applications such as Raman spectroscopy,^3,4^ local chemical reactions,^3^ photocurrent generation,^5,6^ Purcell effect,^7^ and nonlinear optical effects,^8^ while also leading to emerging phenomena including strong light–matter coupling^9−12^ and few-molecule optomechanics.^13,14^ Classical theory predicted ever-enhanced plasmonic capacitive coupling with a decreasing gap size until zero. Nevertheless, as the size of plasmonic nanogaps approaches the nanometer scale, quantum effects can profoundly influence the plasmonic coupling.^15−18^ To understand such influences, intensive research efforts were devoted to various binary plasmonic systems, including NP dimer,^1,19−25^ NP on mirror,^26,27^ and core–shell structures.^28,29^ In general, it was found that the nonlocal screening and electron tunneling effect compromised the plasmonic capacitive coupling, thus limiting the maximum achievable local field enhancement. The details of plasmonic near-field coupling depend on multiple factors, such as nanogap conductance,^21,27^ nanogap morphology,^1,20,22,24^ NP size,^25^ as well as the order and symmetry in the positional arrangement of the NPs.^20,30^ Open discussion continues in the field, urging further efforts from both theoretical and experimental perspectives.^31,32^

In particular, a recent theoretical study suggested a substantially reduced threshold of such quantum limit in a two-dimensional (2D) square-superlattice of plasmonic NPs, in contrast with the binary system.^33^ Compared to isolated binary systems, 2D plasmonic superlattices with uniform and reproducible high-density subnanometer gaps can also provide great benefits for applications in the field of optoelectronics, surface-enhanced Raman scattering (SERS), and optical metasurfaces.^18,34−37^ However, although desired for advanced fundamental understanding and practical applications, experimental demonstration of extended plasmonic superlattices with tunable subnanometer gaps, hence tunable interparticle coupling, remains yet an untackled task. On the one hand, controlled fabrication of nanogaps with subnanometer dimensions remains challenging with lithographic approaches.^2^ On the other hand, there is a trade-off in NPs self-assembling between their long-range order, which often requires long capping ligands to provide enough steric barrier, and the small interparticle gap distances desired.^38,39^ Previous studies have already demonstrated 2D gold nanoparticle (GNP) superlattices with the interparticle gap distance beyond 1 nm,^37,40−44^ as well as GNP monolayers with subnanometer gaps but still of significant gap size variation and/or limited long-range order in NP arrangement.^34,45,46^ In the latter case, defects and broken symmetry in GNP superlattices can lead to compromised near-field plasmonic coupling and deteriorated collective properties.^30,46,47^

A possible approach to achieve 2D NP superlattices with tunable (sub)nanometer gaps is a post-treatment of free-floating self-assembled superlattices at liquid–air interface via ligand exchange.^45,48^ As the interparticle nanogaps are modified, the translational and rotational freedom of NP movement at the liquid–air interface can preserve the NP arrangement. However, current methods of free-floating ligand exchange face inherent limitations. First, the type of ligand available for exchange is restricted by a fixed subphase, resulting in limited tunability of the nanogaps. Second, a subphase ideal for self-assembly can be problematic for ligand exchange or film transfer and vice versa. Diethylene glycol (DEG) has been widely adopted as the subphase to produce various NP superlattices via their evaporation-driven interfacial self-assembly, where size of single domains exceeded one micrometer.^11,37,41−43,48−50^ However, the nonvolatile nature of DEG imposed a challenge on further applications of NP superlattices, where a subsequent long-time drying process in a high-vacuum chamber is often required to remove DEG residuals after NP film transfer.^43,48,49^ Such a long-time drying process can even harm the quality of NP films.^51^ Especially when conducting the ligand exchange, additional chemicals are present in the subphase residual, which will cause uncontrolled local modification of the superlattices as reactions continue during the drying process, leading to inhomogeneity in NP films. Using a volatile subphase like acetonitrile would however compromise the self-assembly process and limit size of single domains to 100–200 nm.^45^

In this work, we obtained large-area (∼1 cm^2^) self-assembled GNP superlattices with oleylamine (OAm) capping via interfacial self-assembly on DEG. We introduced an intermediate subphase exchange process to overcome aforementioned obstacles. This subphase exchange process coordinated the conflicting demands in self-assembly of NP superlattices, subsequent ligand exchange, and film transfer for further applications. Our subphase exchange process also expanded the library of molecules that can be used for ligand exchange, e.g., from various organothiols to inorganic S^2–^, and choices of substrate materials; for instance, soft organic substrates may be accommodated using water as the subphase. Furthermore, the controlled subphase exchange process improved our capability to control the ligand exchange reactions precisely. Such improvement was manifested by an innovative two-step ligand exchange process that fostered cross-linking of short benzenedithiol (BDT) ligands between GNPs. Our precise control over the nanogaps in GNP superlattices allowed active engineering of their electronic transport properties and interparticle plasmonic coupling, hence optical properties. *In situ* reflectance measurements during the ligand exchange process showed gradually red-shifted LSPR peaks with increasing intensity accompanying the diminishing interparticle gap distance until 0.1 nm, which indicates continuously enhanced interparticle plasmonic capacitive coupling in GNP superlattices. This is in contrast to the intensively studied binary systems, for example GNP dimers^23,25^ as well as core–shell structure,^29^ but agrees with the trend suggested by the theoretical study on plasmonic NP array.^33^ The optical response of our GNP superlattice shows unambiguous dependence on subtle changes in nanogaps via the molecular constituents and their conformation, counter to the previous report based on disordered GNP monolayers.^46^ With that, we further demonstrated the application of GNP superlattices as metasurfaces of a tunable effective refractive index. The strong EM field confinement we achieved in high-density subnanometer gaps resulted in the maximal real part of their effective refractive index reaching 5.4 at 783 nm wavelength, exceeding the record values of 5.0.^46,100^

## Results and Discussion

### Fabrication and Structural Characterization of GNP Superlattices

**Figure 1:**
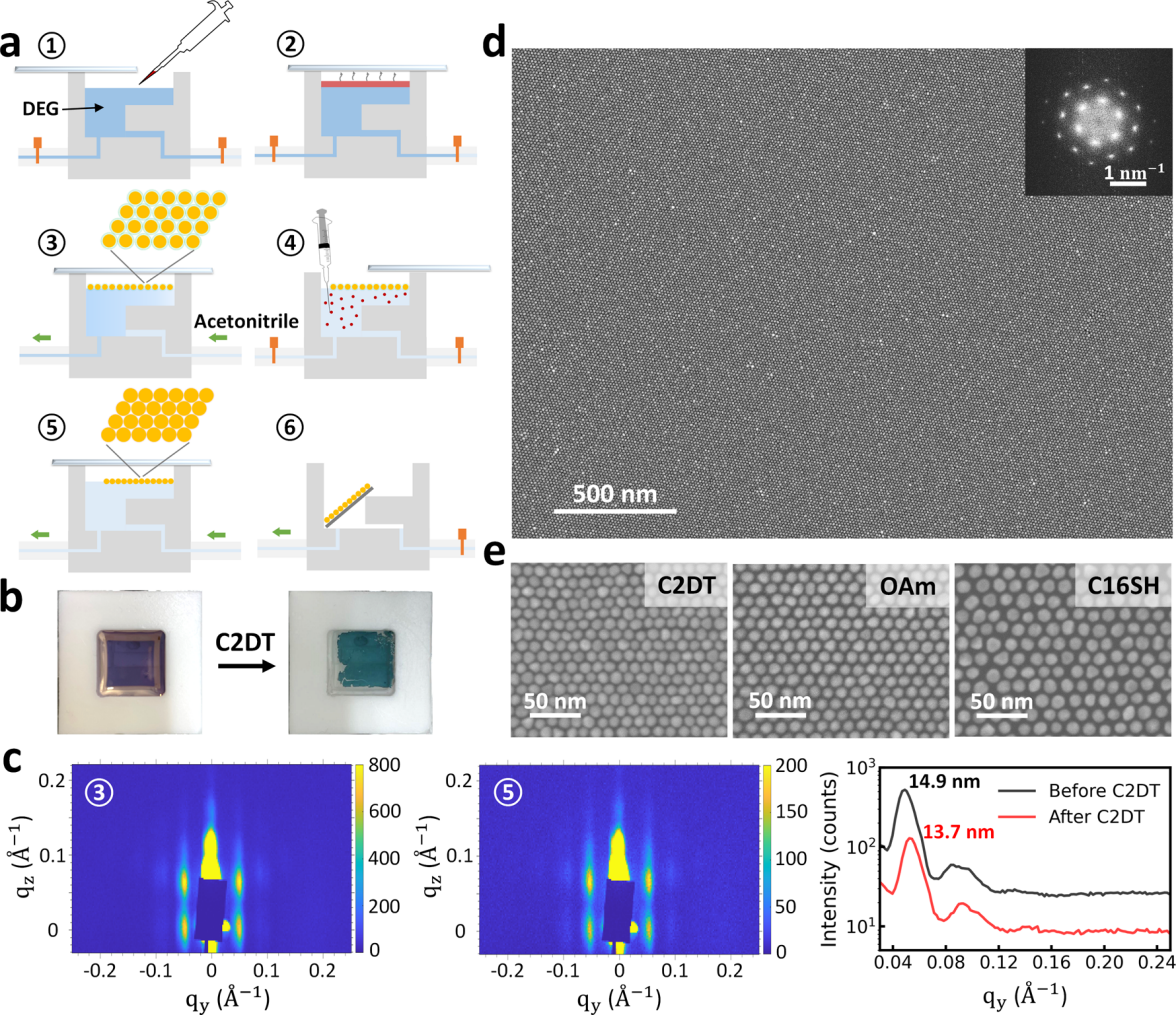
(a) Schematic illustration of ① and ② the GNP superlattices growth, ③ and ⑤ the subphase exchange, ④ the *in situ* free-floating ligand exchange, and ⑥ film transfer process. (b) Photographs of a typical GNP superlattice film subject to ligand exchange with C2DTs. (c) GISAXS patterns measured *in situ* on a free-floating GNP superlattice film before and after ligand exchange with C2DTs, together with the respective line cuts along the horizontal axis. (d) SEM image of a GNP superlattice deposited on SiO_2_/Si wafer after ligand exchange with C2DTs (insert, a two-dimensional fast Fourier transform (2D FFT) power spectrum corresponding to a region of 2 by 2 μm^2^ at the upper left corner of the SEM image). (e) High-resolution SEM images of GNP superlattices with different capping ligands.

A sketch of our fabrication process of the GNP superlattices is presented in Figure 1a. First, we drop cast colloidal solution of GNPs, OAm capped in toluene, on the DEG subphase. The Teflon trough was then closed by a glass lid, allowing slow evaporation of toluene that drove the self-assembly of GNPs. As a result of system entropy maximization, GNP superlattices were formed at the liquid–air interface by the end of the toluene evaporation.^38,42,49^ After the formation of the GNP superlattice, we exchanged the nonvolatile DEG subphase with volatile acetonitrile through a fluidic system. Subsequently, ligand exchange in the GNP superlattices from the OAm to different cappings was conducted by injecting excessive target molecules into the acetonitrile. The reactions during ligand exchange were terminated by a subphase exchange with clean acetonitrile again. Using acetonitrile as the subphase, we obtained large-scale (∼1 cm^2^) high-quality GNP superlattices with subnanometer gaps that can be easily drain-deposited on a solid substrate without the necessity of an extended-period drying process (Supporting Information Figure S1).

Figure 1b shows the typical behavior of a free-floating GNP superlattice film after the self-assembly process, corresponding to Figure 1a ③, and after phase transfer from DEG to acetonitrile and then ligand exchange with 1,2-Ethanedithiol (C2DT), corresponding to Figure 1a ⑤. The film remained macroscopically intact through such process. The isotropic film shrinking was a direct consequence of the nanoscopic reduction of the interparticle distance, while the apparent color change of GNP film from purple to blue reflected the enhanced interparticle plasmonic coupling. We conducted *in situ* grazing-incidence small-angle X-ray scattering (GISAXS) measurements to investigate a real-time change in the nanoscopic arrangement of GNP films. GISAXS measurements provide high accuracy in determining interparticle gap distances with robust statistics thanks to the large-area sampling nature.^42,45,52−56^Figure 1c shows a GISAXS pattern of a free-floating GNP film on acetonitrile before and after the ligand exchange with C2DT, respectively. Due to the low absorption of the X-ray in acetonitrile, we could observe the scattering pattern of both the reflected and transmitted beams.^57^ Such scattering patterns corresponded to hexagonal close-packed monolayer superlattices. The sharp scattering pattern indicated long-range GNP order over large areas.^52^ Line cuts were made along the *q*_*y*_ axis and integrated over the *q*_*z*_ axis of the 2D GISAXS patterns (Figure 1c). From the line cuts we calculated the distances between the center of neighboring GNPs, defined as the interparticle distances, *D*_p–p_ = .^58^ Before ligand exchange, we measured *D*_p–p_ = 14.9 ± 0.1 nm (averaged over three GNP films). By subtracting the size of the Au core of GNPs measured via small-angle X-ray scattering (SAXS), we found the average interparticle gap size, *D*_gap_, of 1.4 ± 0.1 nm for the OAm capped GNP superlattices on acetonitrile. After ligand exchange with C2DT, *D*_gap_ was reduced to 0.2 ± 0.1 nm, similar to the previously reported value.^45^

Besides X-ray measurements, the deposited GNP superlattices were characterized by using a scanning electron microscope (SEM), as shown in Figure 1d and e. Extended hexagonal close-packed monolayer GNP superlattices were confirmed. The typical size of a single domain is several micrometers. Individual domains exceeding 10 μm in size were also observed (Supporting Information, Figure S2). The microscopic arrangement of GNPs remained largely intact after drain-deposition, indicating considerable mechanical robustness of the superlattices and benignity of our method. When probed at high magnifications, modification of *D*_gap_ due to the ligand exchange process was apparent in the SEM images (Figure 1e).

**Figure 2:**
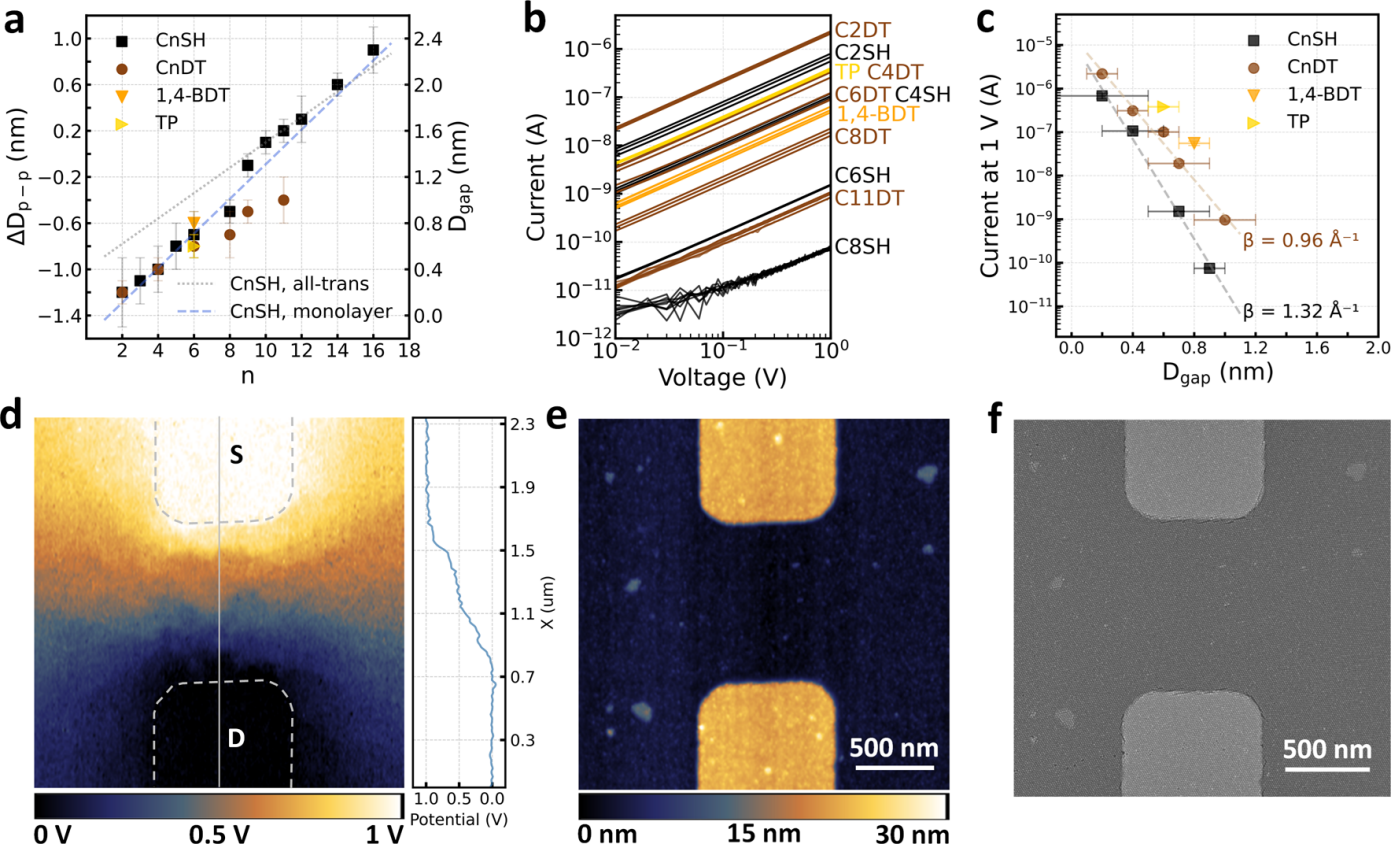
(a) Interparticle distance change, Δ*D*_p–p_, and gap size, *D*_gap_, in GNP superlattices as a function of the alkyl chain length (number of carbon atoms, *n*), including also results of 1,4-BDT and thiophenol (TP) for comparison. (b) Current–voltage curves measured on GNP superlattices subject to ligand exchange with various molecules, each with three repetitions on three different devices. (c) Dependence of the current flow at 1 V on the interparticle gap size in GNP superlattices. The dashed lines indicate exponential fits. (d) Surface potential map and the corresponding line cut obtained from the frequency-modulated Kelvin probe force microscopy scan of an active device based on GNP superlattice after ligand exchange with C2DT. A DC bias of 1 V was applied between the source (S) and drain (D) electrodes. (e) The simultaneously obtained topography map. (f) SEM image of the same device.

Figure 2a shows the dependence of *D*_p–p_ and *D*_gap_ on the alkyl chain length, i.e., the number of carbon atoms (*n*). *D*_p–p_ of the deposited GNP superlattices was quantified by high-resolution SEM images. We found good agreement between the SEM and GISAXS measurements (Supporting Information Figure S3). A 0.3 nm reduction of *D*_p–p_ occurred due to subphase exchange from DEG to acetonitrile. The initial *D*_gap_ in self-assembled GNP superlattices on DEG was thus 1.7 nm, similar to the previously reported values.^39,41^ When OAm ligands were exchanged to alkyl-monothiols, CnSHs, we found a good agreement of our data with the previously reported linear fitting of the thickness of monolayer CnSHs on Au, *t* = 0.15*n* – 0.19 nm, indicated by the dashed blue line.^59^ This suggests interdigitation of alkyl chains on the neighboring particles, as observed in other studies.^40,60−63^

Furthermore, we compare our data with the theoretical length of all-trans-ordered alkyl chains tilted 30° from the surface normal, *l* = 0.11*n* + 0.4 nm, indicated by the gray dotted line.^59,64^ A good match was found for long alkyl-monothiols (9 ≤ *n* ≤ 16), while short alkyl-monothiols led to *D*_gap_ smaller than the theoretical prediction. Such deviation can be attributed to variation in alkyl chain conformations, where more significant chain disorders were observed in shorter chains.^40,63^ In the case of short alkyl-dithiols, CnDTs (*n* < 8), we observed a similar dependence as CnSHs, but with a narrower distribution. As the alkyl chain length exceeded six carbons, a growing discrepancy of *D*_gap_ emerged between the CnSH and CnDT cases. The smaller *D*_gap_ induced by longer CnDTs can be attributed to partial chain loop formation, where both thiol head groups of a CnDT molecule were bound to the same GNP surface. Such loop formation has been shown to become significant as *n* exceeded 8, and remained negligible otherwise.^65^

### Electrical Transport and Scanning Probe Characterization of GNP Superlattices

The tunable *D*_gap_ and composition of the nanogaps provided access to controllable modifications in the electronic coupling between neighboring GNPs. We conducted current–voltage (*I*–*V*) measurements to understand the charge transport behavior across the nanogaps and study their collective behavior in GNP superlattices. The GNP superlattices were deposited on lithography patterned Au electrodes with a separation of 1 μm between the drain and source. Figure 2b shows the *I*–*V* curves measured on GNP superlattices with different capping ligands. The noise level of our current measurement was less than 10 pA. All *I*–*V* curves exhibited a linear (ohmic) dependence. Such behavior is expected for the coherent nonresonant tunneling transport through alkyl thiols or oligophenylene thiols in the low-bias regime.^66−71^

Figure 2c shows the *D*_gap_-dependent currents measured at a 1 V applied bias. At the same interparticle distance, the nanogaps with alkyl-dithiols exhibit higher conductance than their counterparts with alkyl-monothiols. Such observation agrees with the previously reported electrical transport measurements on molecular junctions based on alkyl-monothiols and -dithiols, where despite the similar tunneling barrier through the alkyl chains, dithiol junctions exhibited significantly higher conductance due to their contact resistance 1 to 2 orders of magnitude lower than the monothiol counterparts.^67,70^ Such lower contact resistance in the dithiol case is due to their stronger molecule–electrode coupling as a result of the two thiol end groups binding to gold.^67,70^ An exponential attenuation of current with increasing *D*_gap_ was observed, in accord with the simplified Simmons equation for the low bias regime.^72^ In such case, the current density across the molecule ensembles would follow *J*(*V*) = *J*_0_(*V*)exp(−β*D*), where *J*_0_ is the effective current density at contacting junctions, *V* is the applied bias across the junctions, β is the tunneling attenuation factor, and *D* is the length of junctions.^63,69,73−75^ β depends on the structure of the tunneling barrier and characterizes the efficiency of the tunneling activity. More efficient tunneling corresponds to lower β values. By exponential fitting of *I* as a function of *D*_gap_, we obtained β = 1.32 Å^–1^ for CnSH, and β = 0.96 Å^–1^ for CnDT. Previously reported β values for CnSH on Au were 0.9–1.1 Å^–1^,^66−68,70,73^ and for CnDT were 0.8–0.9 Å^–1^.^67,70,76^ The larger β value here obtained on CnSH can be attributed to the presence of through-space component to the tunneling pathway,^74^ which was largely avoided in the CnDT case, as single CnDT molecules can form a covalent bond on both sides of the nanogaps.

To understand the nanoscopic charge transport properties of our devices, we conducted frequency-modulated Kelvin probe force microscopy (FM-KFM) scans on the active devices. FM-KFM is a noninvasive technique that provides information on the local surface potential with nanoscale spatial resolution.^77,78^ The surface potential map of a C2DT capped GNP superlattice with a 1 V applied bias is shown in Figure 2d, while Figure 2e shows the simultaneously obtained topography image. SEM image of the same device is in Figure 2f. Small voltage drops on single junctions were confirmed from the surface potential map. The continuous potential drop around the periphery of the Au electrodes indicates limited contact resistance. A distinct terracelike drop from source to drain appeared in the surface potential landscape. A previous study suggested that such terraces formed as a result of limited current pathways in the conductive percolation network, where dead ends acquired the potential of their only source node in the spanning cluster.^79^ Interconnections in the spanning cluster can be enhanced when the conductance of junctions increases and when the variance in the conductance of junctions decreases. In terms of junction conductance, exchanging the capping ligands from OAm to C2DT has already resulted in a smoothed surface potential landscape (Supporting Information Figure S4). Given the exponential dependence of junction conductivity on *D*_gap_, any variation in *D*_gap_ will inevitably cause a variation in the junction conductance. In the ideal case, a GNP superlattice with uniformly sized highly conductive nanogaps will lead to a smooth potential transition from the source to the drain. Compared to alkyl chains, the phenyl ring with conjugated carbon bonds of delocalized electrons can provide a higher molecular conductance. We will describe below how such a scenario was approached by a precisely controlled two-step ligand exchange process with an aromatic molecule, benzene-1,4-dithiol (1,4-BDT).

### Two-Step Ligand Exchange

The fabrication of self-assembled GNP arrays with benzenedithiol capping and demonstrated interparticle cross-linking was known to be challenging.^46,80^ When used for ligand exchange here, 1,4-BDT led to a *D*_gap_ of 0.8 nm (Figure 2a), larger than the previously reported values, 0.6–0.7 nm, for self-assembled monolayers (SAMs) of 1,4-BDT.^21,28,81^ The degree of GNP cross-linking by 1,4-BDT was explored by *I*–*V* measurements. The GNP superlattice with 1,4-BDT capping exhibited a conductivity lower than that with TP capping (Figure 2c). In contradiction, previously reported cross-linked molecular junctions based on SAM 1,4-BDT exhibited higher conductance than that of TP.^71^ The larger *D*_gap_ together with a gap conductance lower than that of reported SAM 1,4-BDT thus suggested the formation of partially intercalating SAMs of 1,4-BDT in our nanogaps. Compared to alkyl chains, the phenyl ring of 1,4-BDT is more rigid and bulky,^82^ and the π–π stacking interaction between phenyl rings is stronger than the van der Waals interactions between alkyl chains.^83^ Thus, during their self-assembly process on metal surfaces, the precedently bound 1,4-BDT molecules would impose a steric obstruction to subsequently arriving molecules. Such steric obstruction limits their arrangement to be as close as that of alkyl chains and formation of a herringbone structure is energetically favored for 1,4-BDT molecules.^82−84^ During our standard ligand exchange process, when binding to the GNP surfaces, the chance that phenyl rings on opposite sides of the nanogaps adopted different orientations was high. Due to the strong π–π stacking interaction and steric obstruction, rearrangement of 1,4-BDTs for complete interdigitation was more difficult than alkylthiols, as also observed in previous studies.^73,80^

**Figure 3:**
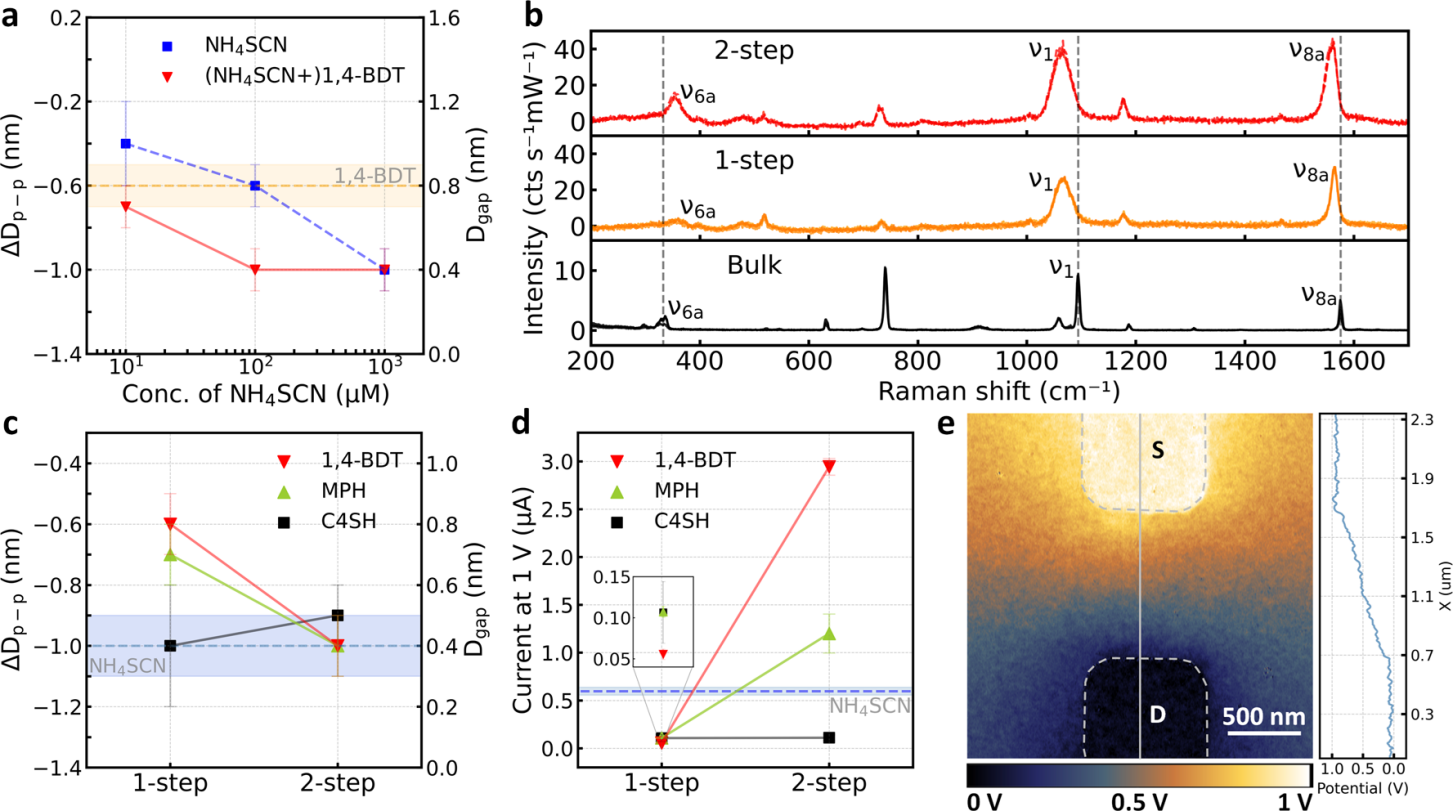
(a) Interparticle distance change, Δ*D*_p–p_, and gap size, *D*_gap_, in GNP superlattices as a function of the NH_4_SCN concentration used in the first step of the two-step ligand exchange process. The dashed orange line indicates the result obtained from a one-step ligand exchange process using 1,4-BDT. (b) SERS spectra on GNP superlattices after two-step and one-step ligand exchange with 1,4-BDT and Raman spectra on bulk 1,4-BDT (powder). The interparticle distance change and gap size in GNP superlattices (c) and currents measured with 1 V bias applied to the GNP superlattices (d) after one-step or two-step ligand exchange process with various ligands. The dashed blue lines in (c) and (d) indicate results obtained from samples after a one-step ligand exchange process with NH_4_SCN. (e) Surface potential map and the corresponding line cut obtained from the FM-KFM scan of a GNP superlattice after two-step ligand exchange with 1,4-BDT. A DC bias of 1 V was applied between the source (S) and drain (D) electrodes.

To facilitate the GNP cross-linking via 1,4-BDT molecules, i.e., the formation of SAMs of 1,4-BDT in the nanogaps, we postulated that a small initial *D*_gap_ at the beginning of ligand exchange would be beneficial. When the initial *D*_gap_ is small enough, due to the spatial confinement, the precedently bound 1,4-BDT molecules in the nanogap would force the subsequently arriving molecules around them to adopt an energetically favorable conformation, regardless of which side of the nanogap they first bind to. Thus, the situation that 1,4-BDT molecules on opposite sides of a nanogap adopt random orientations, creating large energy barrier for their interdigitation, can be avoided. To test our hypothesis experimentally, we developed a two-step ligand exchange process. In the first step, *D*_gap_ was reduced by controlled partial ligand exchange with a short ligand SCN^–^ of different concentrations (Figure 3a).^85,86^ A subphase exchange process was carried out subsequently to remove excess molecules. In the second ligand exchange step, 1,4-BDT molecules were injected into the subphase.

As shown in Figure 3a, when *D*_gap_ at the beginning of the second step was comparable to or smaller than 0.8 nm, corresponding to the one-step 1,4-BDT ligand exchange process, the final *D*_gap_ after the second step was reduced to 0.4 nm. This value is smaller than the previously reported values of SAM 1,4-BDT junctions (0.6–0.7 nm).^21,28,81^ The smaller *D*_gap_ value obtained here is likely due to the lower coverage density of the 1,4-BDT molecules within the nanogaps, corresponding to larger tilting angles of the molecules.^84^

Raman spectra of deposited GNP films after the one- and two-step ligand exchange with 1,4-BDT are presented in Figure 3b. In comparison with the 1,4-BDT powder sample, significant red-shifts of the ring breathing mode (ν_1_, 1094 cm^–1^) and C=C stretching mode (ν_8a_, 1576 cm^–1^) can be observed in GNP superlattices after one-step (ν_1_, 1067 cm^–1^, ν_8a_, 1564 cm^–1^) and two-step ligand exchange (ν_1_, 1063 cm^–1^, ν_8a_, 1556 cm^–1^), respectively. Such red-shifts can be attributed to charge transfer between the chemically bound molecules and the metal, which weakened molecular bonds.^28,87^ The more significant red-shift after two-step ligand exchange hence suggested enhanced interlinking of 1,4-BDT between GNPs. In contrast to the ν_1_ and ν_8a_ modes, a blue-shift of the deformation coupled C–S stretching mode occurred form the 1,4-BDT powder sample (ν_6a_, 333 cm^–1^) to GNP superlattices after one-step (358 cm^–1^), and two-step ligand exchange (354 cm^–1^). This blue-shift is related to dissociation of the S–H bond and formation of the S–Au bond.^87^ In addition to the peak shifts, the broadening of the Raman peaks can be observed in GNP superlattices. The full width at half-maximum (fwhm) of the ν_1_ and ν_8a_ modes are 5.5 cm^–1^ in 1,4-BDT powder, which increased to 26.1 and 13.5 cm^–1^, respectively, in GNP superlattices after one-step ligand exchange, and to 33.8 and 22.5 cm^–1^, respectively, after two-step ligand exchange. The broadening of the Raman peaks is a signature of spatial heterogeneity of the charge transfer effect upon chemisorption. The fluctuation of local Fermi energies of GNPs and the different conformations of 1,4-BDT molecules lead to different red-shifts, hence the broadening of Raman peaks.^28^ The influence of such spatial heterogeneity becoming more significant in the two-step ligand exchange case suggests a stronger charge transfer effect. Moreover, the Raman peak intensity of GNP superlattices after two-step ligand exchange was further enhanced compared to that of one-step ligand exchange. Again, such increased SERS intensity can be attributed to the larger charge transfer enhancement, stronger local EM field enhancement, or a combination of both.^3,28^

As a control group, 4-mercaptophenol (MPH) and C4SH, both of similar molecular length as 1,4-BDT, were employed for ligand exchange. Figure 3c shows the corresponding values of *D*_gap_ after different ligand exchange processes, where 1 mM NH_4_SCN was always used in the first step of the two-step ligand exchange. When conducting a two-step ligand exchange for the aromatic molecule MPH, *D*_gap_ can be further reduced from the one-step case, similar to 1,4-BDT. In contrast, when using C4SH, the difference in the *D*_gap_ between the one- and two-step ligand exchange processes was insignificant compared to the measurement uncertainty. Figure 3d compares the electrical transport properties of different GNP films, where nanoscopic variations in the molecular conformation became discernible. When C4SH was used, similar gap conductance was observed after a one- or two-step ligand exchange process. In contrast, when aromatic molecules were used, *D*_gap_ was further reduced via the two-step ligand exchange process and a significant increase in the nanogap conductance was observed. Moreover, 1,4-BDT led to more conductive nanogaps than MPH, demonstrating the increased GNP cross-linking by 1,4-BDT molecules when performing two-step ligand exchange. Again, we performed an FM-KFM scan on an active GNP superlattice device after a two-step ligand exchange with 1,4-BDT. As shown in Figure 3e, a rather smooth transition from source to drain was observed in the potential map (*cf*. Supporting Information Figure S5 for the corresponding topography images). In such a scenario, current flowed homogeneously across the GNP network with many interconnected conductive paths.^79^

### Ligand Exchange to Inorganic S^2–^

**Figure 4:**
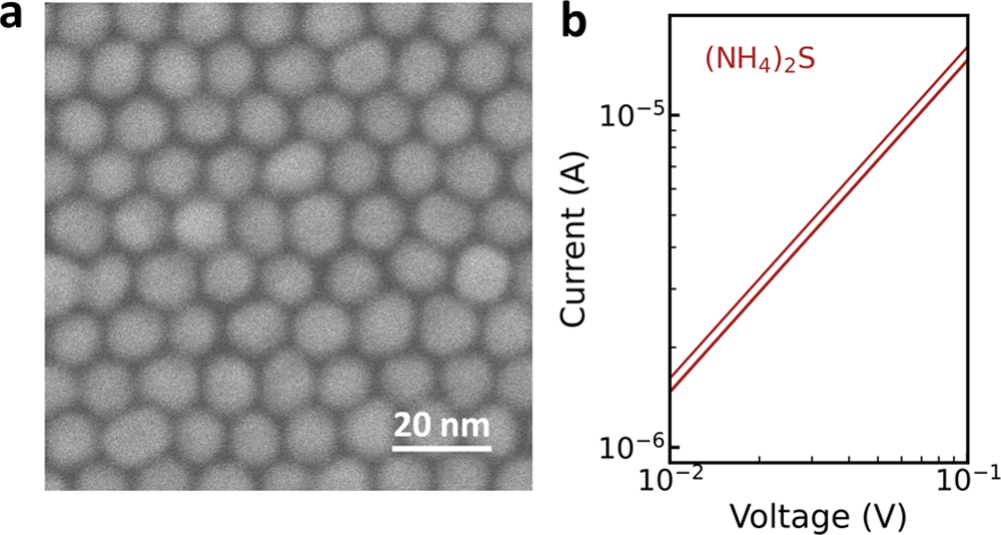
(a) High-resolution SEM image of a GNP superlattice after ligand exchange with (NH_4_)_2_S. (b) Current–voltage curves measured on GNP superlattices after ligand exchange with (NH_4_)_2_S, with three repetitions on each of the three different devices.

Compared to organothiol molecules, inorganic surface ligands were shown to introduce stronger electronic coupling between NPs.^39,88,89^ Taking advantage of the versatility of our method in choosing compatible ligands and subphases, we explored the use of S^2–^ for ligand exchange. In such case, a subphase of mixed *N*,*N*-dimethylformamide (DMF) and *N*-methylformamide (NMF) was employed (2:1 in volume ratio). In our experiment, mixing DMF and NMF was crucial for obtaining well-ordered GNP superlattices through the ligand exchange process. While NMF can stabilize (NH_4_)_2_S in the subphase, DMF prevented GNPs from losing from superlattices into the subphase after surface capping by S^2–^. DMF is also known to promote the displacement of ligands from the NP surfaces.^51,90^ When 40 mM (NH_4_)_2_S was used for ligand exchange, we obtained GNP superlattices with *D*_gap_ = 0.1 ± 0.1 nm. A high-resolution SEM image of the GNP superlattice is shown in Figure 4a (Supporting Information Figure S6 for more SEM images). The intrinsic variations in the shape and size of our synthesized GNPs caused corresponding nonuniformities in the size and morphology of nanogaps at this touching limit.

*I*–*V* curves measured on the GNP superlattices are displayed in Figure 4b. The conductivity of GNP superlattices increased by ca. 2 orders of magnitude compared to that after ligand exchange with C2DT or 1,4-BDT (two-step). The vanishing nanogaps with S^2–^ capping resulted in the strongest electronic coupling between GNPs. The conductivity of GNP superlattices with S^2–^ capping is estimated as 1.4 × 10^4^ S/m, comparable to the previously reported values (1.1–1.7 × 10^4^ S/m) for three-dimensional GNP superlattices with metal–chalcogenide complex (chalcogenidometallate) ligands.^39^ Note that a maximum voltage of 0.1 V was applied here to the GNP superlattices to prevent GNP damage caused by Joule heating.

### *In Situ* Optical Reflectance Measurements on GNP Superlattices during Ligand Exchange

**Figure 5:**
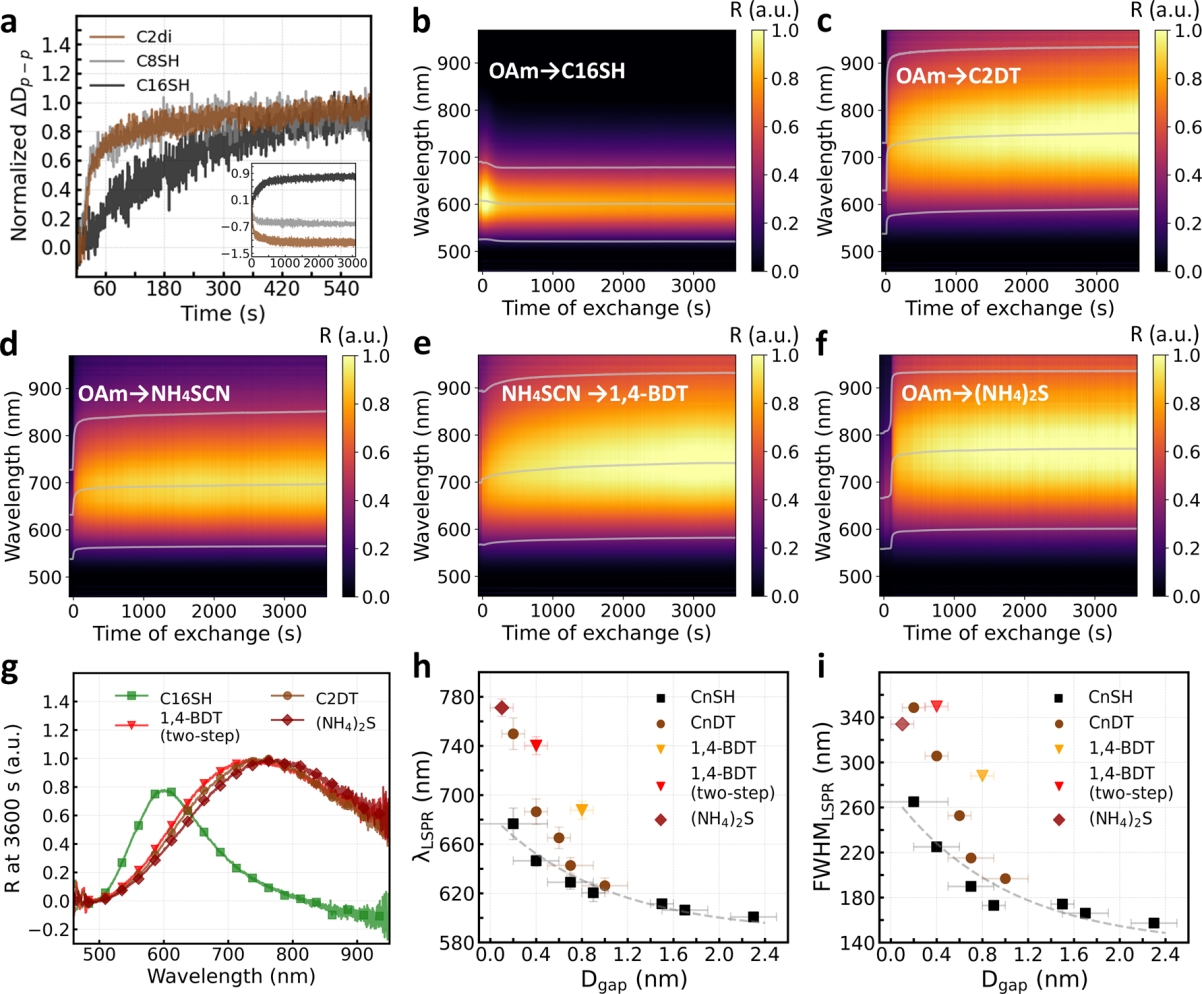
(a) *In situ* time evolution of normalized change in interparticle distance during different ligand exchange processes, measured by GISAXS (inset, the corresponding time evolution of absolute interparticle distance). The *in situ* optical spectra of normalized reflectance (R) measured on free-floating GNP superlattices during ligand exchange with C16SH (b), C2DT (c), NH_4_SCN (first step) (d), 1,4-BDT (second step) (e), and (NH_4_)_2_S (f). The plasmonic resonance peak position and fwhm are indicated by the superimposed line plots. Normalized reflectance spectra (g), plasmonic resonance peak wavelength λ_LSPR_ (h), and fwhm of the plasmonic resonance peak (i) measured on different free-floating GNP superlattices after 1 h of ligand exchange. The dashed lines in (h) and (i) indicate exponential fits.

After demonstrating nanogaps with controllable sizes and constituents, we now address their implications in interparticle plasmonic coupling within the GNP superlattices. Compared to measurements commonly conducted on deposited samples, we took advantage of the gradual evolution of nanogaps during our ligand exchange process and monitored the corresponding *in situ* evolution of LSPRs in GNP superlattices via optical reflectance measurements. The time evolution of *D*_p–p_ from *in situ* GISAXS measurements is shown in Figure 5a. After launching ligand exchange, we observed 90% of the *D*_p–p_ variation in the first few minutes despite various molecule lengths. The extended time evolution of *D*_p–p_ can be well-fitted by biexponential functions (Supporting Information Figure S7). Such combination of a fast and a slow process can have two origins: first, the different kinetics of ligand adsorption at low and high coordination number sites,^91^ and second, the fast Langmuir adsorption of ligands followed by their slow conformational rearrangement.^92^*In situ* optical reflectance spectra of GNP superlattices in Figure 5b–e provided far-field information about the LSPRs in GNP superlattices, with statistics from more than a billion of simultaneously probed nanogaps.^33,46,93^ Due to the low level of defects in our GNP films, the observed overall optical behavior should be representative of that in the well aligned superlattices. In accordance with the initial fast evolution of *D*_p–p_, all the *in situ* reflectance spectra, Figure 5b–f, showed a rapidly shifting bonding dipolar plasmon (BDP) mode upon injection of new ligands. Such spectral shifts continued monotonously and approached their steady state after an extended period of measurements. When C16SH was used for ligand exchange, the increased size of nanogaps led to weakened plasmonic capacitive coupling between neighboring GNPs, thus a blue-shifted BDP mode with reduced intensity (Figure 5b). When short ligands were used, plasmonic capacitive coupling was enhanced, reflected in the monotonously red-shifting and broadening BDP mode and its increasing intensity (Figure 5c–e).^1,33,94,95^ Previous studies observed screened BDP mode, thus weakened local field enhancement, with increasing conductive coupling in (sub)nanometer gaps between the adjacent GNPs. Provided high enough gap conductance, BDP mode was replaced by emerging blue-shifting charge transfer plasmon (CTP) modes.^17^ Our observation of the monotonously red-shifted and enhanced BDP mode in GNP superlattices until the touching limit of neighboring GNPs therefore suggests the essential difference in plasmonic near-field coupling between the extended GNP superlattices and previously reported isolated binary systems,^23,26,96,97^ and a GNP monolayer with lower order of arrangement.^46^

We measured the reflectance spectra on floating GNP superlattices after one h of ligand exchange to compare different capping ligands. The spectra corresponding to Figure 5b–f are presented in Figure 5g. Figure 5h shows the extracted peak wavelengths of the BDP mode, λ_LSPR_, of different samples with their fwhm’s given in Figure 5i. λ_LSPR_ showed an angstrom sensitivity to *D*_gap_. Thanks to the relatively large range of achievable *D*_gap_, the dependence of λ_LSPR_ on *D*_gap_ with CnSH capping can be well fitted by an exponential function, λ_LSPR_ = 97.1exp(−*D*_gap_) + 587.1 nm, expanding the applicable regime of “plasmonic nanoruler” to GNP superlattices.^95,98^ Besides, λ_LSPR_ exhibited high sensitivity to the constituents of nanogaps as well. Given the same *D*_gap_ values, the red-shift of the BDP mode increased from CnSH to CnDT, and then 1,4-BDT capping, as conductance and permittivity of the corresponding nanogaps increased. The fwhm of the plasmon peaks varied in a trend similar to that of the peak wavelengths, where their dependence on *D*_gap_ can be fitted by fwhm_LSPR_ = 137.2exp(−*D*_gap_) + 136.1 nm for samples with CnSH capping.

### Metasurfaces Consisting of GNP Superlattices

**Figure 6:**
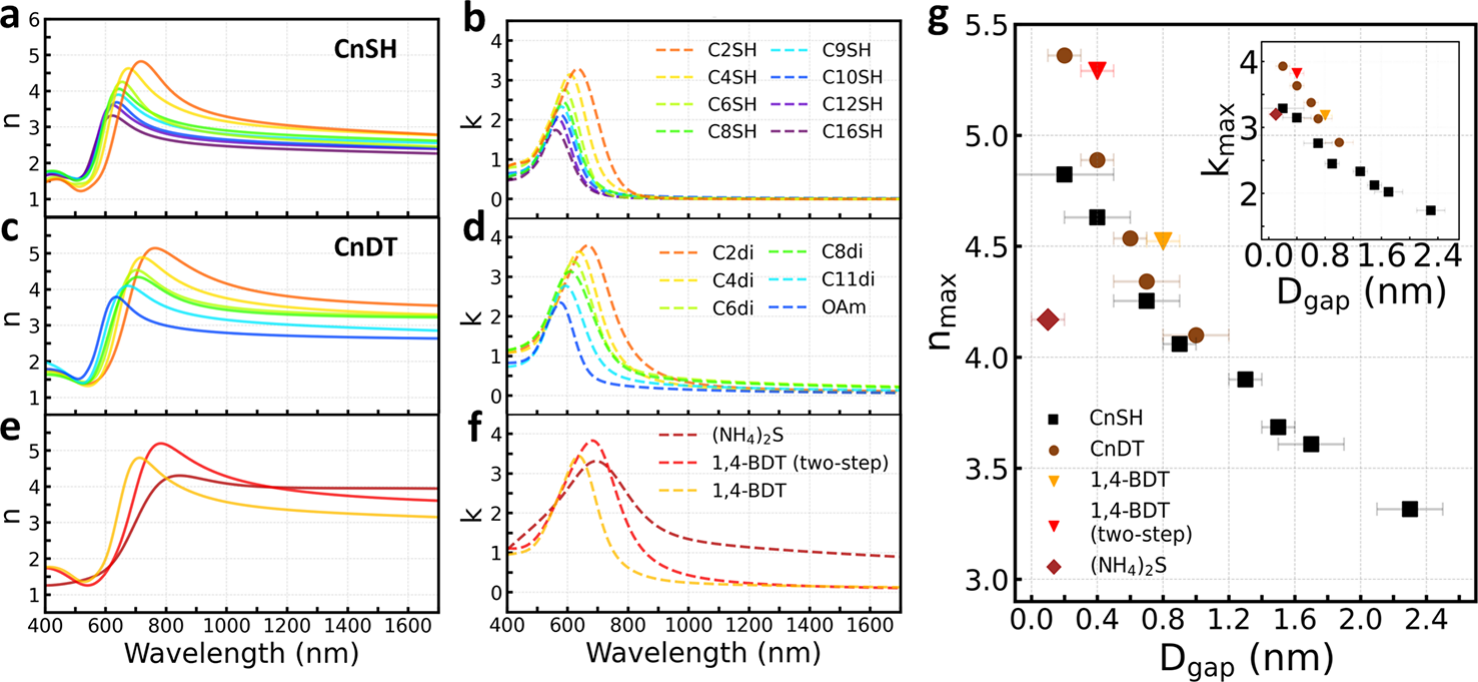
(a–f) The effective refractive index obtained from ellipsometry measurements on different GNP superlattices. (a–e) Real part, *n*, with the corresponding imaginary part, *k*, in (b), (d), and (f), respectively. (g) The dependence of *n*_max_ on interparticle gap distance, *D*_gap_ (inset, the dependence of *k*_max_ on *D*_gap_).

We further explored the application of GNP superlattices as metasurfaces after their deposition on the SiO_2_/Si wafers. Their effective refractive index, both real (*n*) and imaginary (*k*) parts, was obtained by ellipsometry measurements, as shown in Figure 6a–f. Both *n* and *k* showed resonant behavior, indicating their plasmonic nature. Both *n* and *k* varied considerably over broad wavelength ranges between different samples, which can be explained by the decoupled electric and magnetic response in plasmonic GNP superlattices.^99,100^ Through the rational design of the nanogap size and constituent, the plasmonic near-field coupling in our GNP superlattices can be precisely tuned, thus providing an efficient way to achieve large-scale metasurfaces with an engineered refractive index. Specifically, increased field confinement and enhancement in plasmonic nanogaps can induce larger effective refractive index, enabling high-optical-index metamaterials.^36,37,99,100^

As displayed in Figure 6a–f, given a similar gap constituent, both *n* and *k* red-shifted with decreasing *D*_gap_, due to enhanced interparticle plasmonic capacitive coupling. Figure 6g presents the extracted maxima of *n* and *k* from different samples. The maximum of *n*, *n*_max_, and *k*, *k*_max_, increased with decreasing *D*_gap_, or increased gap conductance and permittivity, e.g., from C4SH to C4DT then 1,4-BDT (two-step). For GNP superlattices after ligand exchange to C2DT and 1,4-BDT (two steps), *n*_max_ reached 5.4 ± 0.2 and 5.3 ± 0.2, respectively, around 780 nm wavelength. Our results exceeded the previously reported values of 5.0 measured on metasurfaces of monolayer GNP arrays.^46,100^ Such high values of effective refractive index we obtained here indicate more extreme field confinement achieved in the subnanometer gaps, enabled by the superior order of our GNP arrangement and well-controlled gap constituent. In GNP superlattices after ligand exchange to S^2–^, *n* and *k* exhibited a broadened resonance nature. The *n*_max_ value of 4.2 ± 0.1 was found at 850 nm wavelength and remained as high as 4.0 over a broad range of wavelengths into the near-infrared (NIR) regime. The smaller *n*_max_ value after exchange to sulfide ligands compared with those of C2DT and 1,4-BDT (two-step) indicates weakened local field enhancement in the former case. This can be attributed to the largely enhanced quantum tunneling events in the smallest gaps of sulfide capping, which compromise the plasmonic capacitive coupling.^1,33^ It is worth noting that broadened resonance peak in *k* of nonvanishing value into the NIR regime was observed, indicating the more dissipative nature of the interaction between GNP superlattices and EM excitations in the case of vanishing nanogaps.

## Conclusions

In conclusion, we demonstrated that interfacial self-assembly in combination with subphase exchange and free-floating ligand exchange is an efficient route for scalable production of GNP superlattices of high-density plasmonic (sub)nanometer gaps. The physical properties of the nanogaps, e.g., size, conductance, and permittivity, can be precisely modified by selecting appropriate ligands and suitable subphases for ligand exchange. This allowed active engineering of the nanoscopic and collective electrical transport property of GNP superlattices, and the interparticle plasmonic coupling that governs the optical properties of GNP superlattices. Continuously enhanced interparticle plasmonic capacitive coupling in GNP superlattices with diminishing *D*_gap_ until 0.1 nm was observed during reflectance measurements, indicated by gradually red-shifted LSPR peaks with increasing intensity. When functioning as metasurfaces, the GNP superlattices exhibited a tunable refractive index over a broad range of wavelengths. The high-density plasmonic (sub)nanometer gaps supporting extreme EM field confinement and enhancement led to metasurfaces achieving *n*_max_ of 5.4. GNP superlattices fabricated with such consistently tunable subnanometer gaps are a promising platform for further applications requiring scalable and reliable plasmonic hotspots, such as plasmonic electronics and plasmon-enhanced spectroscopy. Meanwhile, it provides additional chances for fundamental investigation into quantum influence on plasmonic coupling until the touching limit. Our method serves also as a universal solution for fabricating other nanoparticle superlattices of controllable surface capping and interparticle coupling, which can be interesting for a spectrum of practical applications, e.g., superfluorescence,^101,102^ optoelectronics,^103,104^ and spintronics.^105,106^

## Methods

### Chemicals and Materials

All chemicals were used without further purification. Trisodium citrate dihydrate (99%), potassium carbonate (≥99%), oleylamine (OAm, 70%), ethanol (*>*99.8%), acetonitrile (*>*99.9%), *N*,*N*-dimethylformamide (DMF, extra pure), *N*-methylformamide (NMF, 99%), glass syringe (Hamilton 10 mL), ethanethiol (C2SH, 97%), 1-propanethiol (C3SH,99%), 1-butanethiol (C4SH, 99%), 1-pentanethiol (C5SH, 98%), 1-hexanethiol (C6SH, 95%), 1-octanethiol (C8SH, ≥98.5%), 1-nonanethiol (C9SH, 99%), 1-decanethiol (C10SH, 96%), 1-undecanethiol (C11SH, 99%), 1-dodecanethiol (C12SH, ≥98%), 1-tetradecanethiol (C14SH, ≥98%), 1-hexadecanethiol (C16SH, 99%), 1,2-ethanedithiol (C2DT, ≥98%), 1,3-propanedithiol (C3DT, 99%), 1,4-butanedithiol (C4DT, 97%), 1,6-hexanedithiol (C6DT, 96%), 1,8-octanedithiol (C8DT, 97%), 1,9-nonanedithiol (C9DT, 95%), 1,11-undecanedithiol (C11DT, 99%), benzene-1,4-dithiol (1,4-BDT, 99%), thiophenol (TP, ≥99%), 4-mercaptophenol (MPH, 97%), ammonium thiocyanate (NH_4_SCN, 99.99% trace metals basis), and ammonium sulfide solution ((NH_4_)_2_S, 40–48 wt % in H_2_O) were from Sigma-Aldrich. Tetrachloroauric(III) acid trihydrate (HAuCl_4_·3H_2_O, 99.99% trace metal basis) was from Alfa Aesar. Diethylene glycol (DEG, 99%) was from Acros Organics. Toluene (*>*99.5%) was from VWR. Ultrapure water was produced using the Synergy UV water purification system from Millipore and filtered at 0.22 μm. The components for the fluidic system were from IDEX Health & Science, including PFA tubes, PEEK valves and connectors.

### Synthesis of GNPs

Aqueous solutions of citrate-stabilized GNPs was prepared by a modified Turkevich method.^40^ First, 80 mL of chloroauric acid solution (1 mL of 1% w/w HAuCl_4_ in 79 mL of ultrapure water) was mixed with 20 mL of solution of the reducing agents (16 mL of ultrapure water, 4 mL of 4% w/w trisodium citrate, 0.03 mL of 1% w/w tannic acid, and 0.05 mL of 50 mM potassium carbonate) at 90 °C under vigorous stirring. The solution was maintained at 90 °C for 10 min after turning into ruby red, then cooled to room temperature, and finally stored at 5 °C. Dynamic light scattering (DLS) was used to check the volume mean diameter of GNPs, giving a value of 13.2 ± 0.5 nm from measurements on five different batches used in our experiments.

### Phase Transfer of GNPs

Before phase transfer, the GNP solution was centrifuged at 5 °C for 10 min with a relative centrifugal force of 16060*g*. The original aqueous GNP solution was concentrated by redispersing the precipitate into ultrapure water of one-tenth of its original volume. During phase transfer, 1 mL of concentrated GNP solution and 1 mL of ethanol were mixed in a glass vial. Then, 1 mL of a 0.1 M solution of oleylamine in toluene was infused slowly into the glass vial. The mixture was shaken vigorously by hand for 1 min, and left still overnight for complete phase separation.^42^ After the two phases separated, the OAm capped GNPs suspended in toluene could be collected with a pipet.

### Self-Assembling of GNP Superlattices at the Liquid–Air Interface

The monolayer GNP superlattice films were obtained by interfacial self-assembling using the method introduced by Dong et al.^49^ 100 μL of the GNP suspension (OAm capped) was gently spread on the surface of DEG (1.7 × 1.5 cm^2^, volume 1.3 mL) in a Teflon well. Afterward, the well was covered by a glass slide to slow the evaporation of toluene. The self-assembly process was allowed to proceed overnight, ensuring complete evaporation of toluene. Eventually, a solid golden film formed on the surface of DEG.

### Subphase Exchange

Before free-floating ligand exchange with organic molecules, DEG was exchanged with acetonitrile at a constant rate of 100 μL min^–1^ controlled by a syringe pump (Legato 270, KD Scientific). If (NH_4_)_2_S was desired for ligand exchange, a premixed solvent of DMF and NMF (2:1 in volume ratio) was used to substitute for DEG following the same procedure. The volume of acetonitrile or DMF/NMF mixture used for the subphase exchange process is five times that of the DEG to ensure a thorough exchange. After the free-floating ligand exchange with organic molecules, the subphase was exchanged with 6.5 mL of clean acetonitrile at a constant rate of 200 μL min^–1^. After ligand exchange with (NH_4_)_2_S, the subphase was exchanged with 3 mL of clean DMF/NMF mixture and then 6.5 mL of acetonitrile, both at 200 μL min^–1^.

### Free-Floating Ligand Exchange

For free-floating ligand exchange, a concentrated solution of the target ligands was slowly injected into the subphase by hand with a syringe at one of the well corners.^43^ We used typically 10 mM and 10 μM target molecule concentrations in the subphase during a common exchange and for *in situ* GISAXS measurements, respectively. A lower concentration of the target ligands was used for *in situ* GISAXS measurements, as a slower reaction rate could benefit the capture of more details in time-resolved measurements. When preparing the concentrated solutions, NMF was used for (NH_4_)_2_S, DMF was used for 1-tetradecanethiol, 1-hexadecanethiol, and 1,4-BDT, and all other molecules were dissolved in acetonitrile. The waiting time for ligand exchange was 1 h, with the Teflon well covered by a glass slide. At 10 mM, the number of ligands added for exchange is estimated to be 3 orders of magnitude higher than the number of ligands needed by GNPs at the interface for capping their whole surfaces.^107^ The large excess of new ligands and waiting time of 1 h were set to ensure uniform ligand exchange over the GNP film.

### Drain-Deposition of GNP Superlattices

When the GNP superlattices were transferred to a solid substrate, minimized disturbance to the floating GNP film was exerted by draining the subphase at a slow speed, so that the floating film can adapt to the gradual change of the interface. Meanwhile, to allow free movement of the GNP film, its pinning at the edges of the Teflon well was gently broken by the sharp syringe tip.

### SAXS and GISAXS Characterization

The SAXS and *ex situ* GISAXS characterizations were performed on a custom-designed NanoStar SAXS system. The collimated X-ray beam (Ga Kα line, wavelength = 0.134 nm) was shaped by a pinhole collimator with a diameter of 550 μm. The *in situ* GISAXS characterization was performed in a custom-made laboratory setup. A microfocus X-ray source delivered a focused X-ray beam (Cu Kα line, wavelength = 0.154 nm) with a spot size of 250 μm (fwhm) at a focal length of 56 cm (5 mrad divergence) and a total flux of 3.3 × 10^8^ photons per second. A fast 2D X-ray detector (Pilatus 100 K, Dectris) was employed. To access the GNP film by X-ray beam, the surface of the subphase was raised above the Teflon trough by injecting extra acetonitrile into the subphase (Supporting Information Figure S8). The X-ray grazing-incidence angle was 0.3° with respect to the plane of the liquid surface. The time-resolved GISAXS patterns were collected with a 500 ms resolution. A GISAXS pattern with 10 s integration time was collected before and after the time-resolved measurements. During *in situ* GISAXS measurements, the evaporation of acetonitrile from subphase was compensated by injection at ∼10 μL min^–1^ to maintain a steady liquid surface. The SAXS and GISAXS measurements were calibrated with silver behenate. Further details on the X-ray setup can be found elsewhere.^52,108^

### SEM

The GNP films were deposited on SiO_2_/Si wafers for SEM characterization. We used a Hitachi SU8230 electron microscope, operating at 10 kV. To avoid possible bias due to the microscopic sampling and ensure that the data are representative, the mean values were obtained for each sample from five different spots spanning across a deposited GNP superlattice film to determine the interparticle distances. Quantitative analysis of SEM images was performed using Gwyddion.^109^ We first calculated the 2D autocorrelation function of high-resolution SEM images (200 K magnification) to calculate the interparticle distances. Then the statistical mean value of the nearest-neighbor distance was calculated by fitting the radial distribution function of the 2D autocorrelation function with a Gaussian function.

### Electrical Transport Measurements

The GNP superlattices were deposited on thermally oxidized 300 nm SiO_2_/Si wafers with patterned Au electrodes for electrical conductivity measurements. The electrodes were patterned using electron-beam lithography with metal deposition via a commercial evaporator (Evatec BAK501 LL). The thickness of the electrodes was 23 nm (20 nm Au and 3 nm Ti adhesion layer) at the apex and 83 nm in the remaining part (80 nm Au and 3 nm Ti). The Au electrodes on the SiO_2_/Si wafer were wire-bonded to a chip carrier for handling electrical contacts. The substrates with Au electrodes were cleaned by sonication in acetone, then IPA, for 3 min, respectively, followed by a 3 min ozone treatment right before usage. *I*–*V* curves were acquired under a N_2_ atmosphere at room temperature, with an Agilent B2912 precision source-measure unit. Three different devices were measured on each GNP film to avoid possible bias.

### Scanning Probe Microscopy

Atomic force microscopy (AFM) images were acquired with a Cypher S from Oxford Instruments under a dry air atmosphere and ambient conditions. To improve the electrostatic sensitivity, Pt-coated AC240 cantilevers from an Olympus were used. The scans were performed using frequency-modulated AFM with a net-attractive feedback (frequency shift of ca. −15 Hz, amplitude of ca. 18 nm) at a scan speed of 2.5 μm per second. The topography images were leveled and flattened using Gwyddion.^109^ Simultaneously, we determined the local surface potential of the sample by using FM-KFM with sideband demodulation. A home-built algorithm based on a Kalman filter was used to improve the feedback performance.^77^ Both AFM and KFM controls were performed on an external device (HF2LI) from Zurich Instruments. Further details of the KFM setup may be found elsewhere.^77,78^

### DLS Measurements

DLS measurements were performed with a Zetasizer Nano ZS instrument (Malvern Instruments). The number of runs per measurement was 18. Three measurements were recorded for each sample with standard deviations of the volume means below 1%.

### Raman Spectroscopy

Raman spectra were collected using an NT-MDT Raman system with a 100× objective (NA = 0.8). A red laser (633 nm) was used as the excitation, together with a grating of 600 lines mm^–1^. The exposure time of each spectrum for bulk 1,4-BDT (powder) samples was 10 s and GNP samples 4 s. The measurements on GNP samples were carried out in three random locations spanning across the film to avoid possible bias due to microscopic sampling. The Raman shifts of all spectra were calibrated by a Si peak from the substrate at 520 cm^–1^.

### *In Situ* Reflectance Spectroscopy

The optical reflectance spectra of GNP films were obtained with a home-built setup. A broadband fiber-coupled halogen lamp (OSL2IR, Thorlabs) was used as the light source, which was focused by a 5× objective (Nikon, NA = 0.13) onto the sample. A high-resolution spectrometer (HR4000CG-UV-NIR, Ocean Optics) was used to record the spectra. The measured wavelengths were between 450 and 1000 nm. The dark spectrum was collected by focusing on the empty liquid surface for calibration, while a reference spectrum was collected by focusing on a silver-coated mirror (5103, New Focus). A schematic sketch of the setup can be found in the Supporting Information Figure S9.

### Ellipsometry

The GNP superlattices were deposited onto SiO_2_/Si wafers for ellipsometry measurements. Ellipsometry spectra of the samples were measured by using an M-2000 ellipsometer (J.A. Woollam Co.). The reflection measurements were carried out between 70° and 80° incidence angle. The wavelength varied from 400 to 1700 nm in steps of 10 nm. The values of the complex reflectance ratio were modeled using CompleteEASE software (J.A. Woollam Co.) to determine the effective refractive index of the GNP metasurfaces. A uniform medium was assumed when modeling GNP films since their dimension is in the deep subwavelength regime. The modeled layer was defined as three generic oscillators, each consisting of one Lorentz and one Drude term, along with one offset and two poles outside the collected data.^44,97^ The generic oscillators ensure a Kramers–Kronig consistent line shape. The layer thickness was set to the measured interparticle distance of the GNP films. Representative complex reflectance ratio spectra and modeling results can be found in Supporting Information Figure S10.

## Supporting Information Available

A photograph of the drain-deposited GNP superlattices on a SiO_2_/Si wafer; additional SEM images of the GNP superlattices; a comparison between SEM and GISAXS on interparticle distance measurement; additional FM-KFM images of the GNP superlattices; fitted time-evolution of the interparticle distance change from *in situ* GISAXS measurements; a photograph of the experimental setup for *in situ* GISAXS measurements; a schematic illustration of the optical setup for *in situ* reflectance measurements; the fitted complex reflectance ratio of the GNP superlattices (PDF)
