# Supplementary material for: Tunable Subnanometer Gaps in Self-Assembled Monolayer Gold Nanoparticle Superlattices Enabling Strong Plasmonic Field Confinement
Source: ACS Nano. 2023 Jun 24;17(13):12774–87. doi: 10.1021/acsnano.3c03804 (PMC10339788; doi:10.1021/acsnano.3c03804)
Supplement: Supplementary file 1 — nn3c03804_si_001.pdf [file nn3c03804_si_001.pdf]

Supporting Information for

Tunable Sub-Nanometer Gaps in Self-Assembled Monolayer Gold Nanoparticle Superlattices Enabling Strong Plasmonic Field Confinement

Bin Lu,[*,†] Karol Vegso,[‡] Simon Micky,[‡] Christian Ritz,[†] Michal Bodik,[†] Yuriy Myronovych Fedoryshyn,[¶] Peter Siffalovic,[‡] and Andreas Stemmer[*,†]

†*Nanotechnology Group, ETH Zürich, Säumerstasse 4, CH-8803 Rüschlikon, Switzerland.*

‡*Institute of Physics SAS, Dubravska cesta 9, 84511 Bratislava, Slovakia.*

¶*Institute of Electromagnetic Fields, ETH Zürich, Gloriastrasse 35, CH-8092 Zürich, Switzerland.*

E-mail: lubinlu@ethz.ch; astemmer@ethz.ch

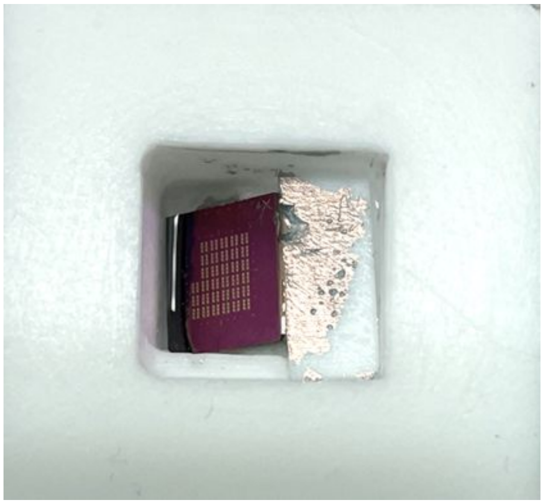

Figure S1. Photograph of GNP superlattices after ligand exchange with 1,4-BDT (two-step), and drain deposition on a SiO_2/Si substrate with patterned Au structures.

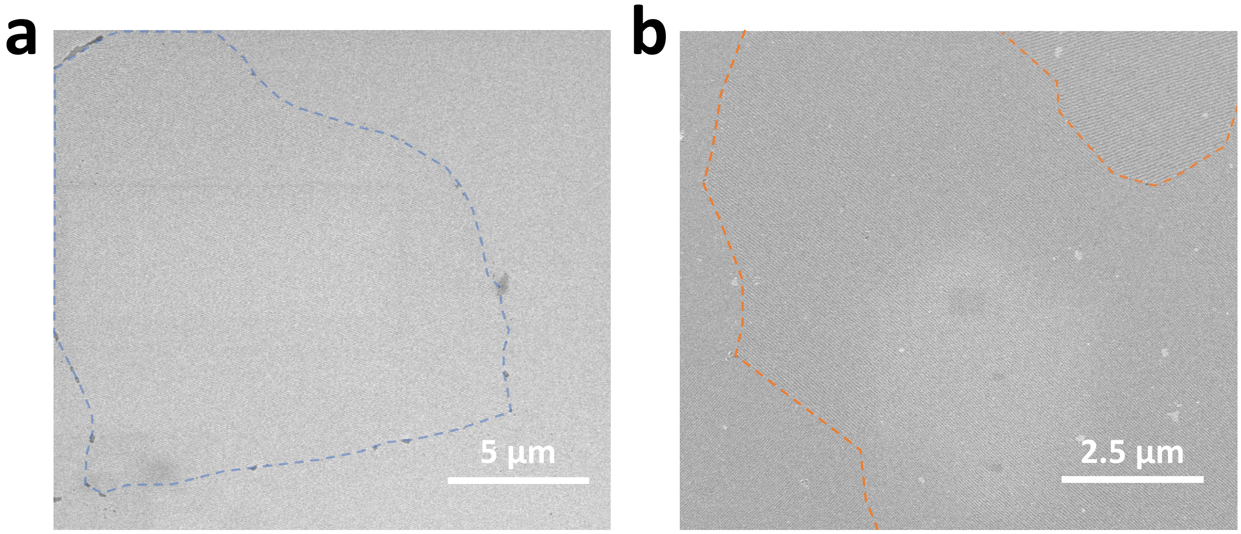

Figure S2. Low magnification SEM images of GNP superlattices with OAm (a), and C2DT (b) as capping ligands. The dashed lines indicate grain boundaries. Moiré fringes formed at these magnifications as a result of highly ordered GNP arangement.[1–3]

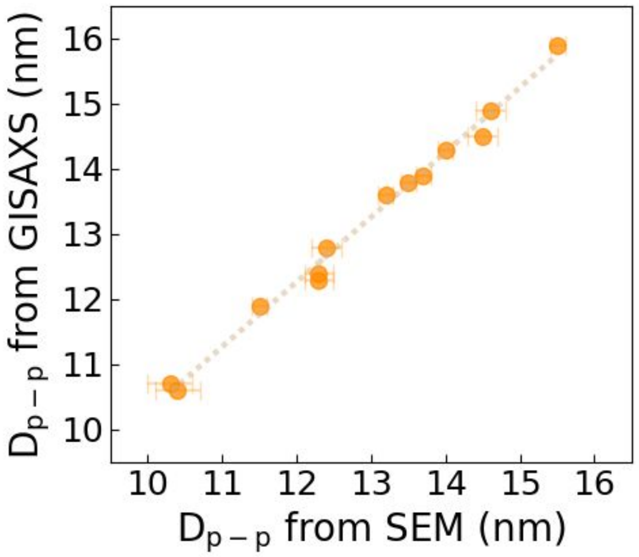

Figure S3. Comparison between the interparticle distances measured by SEM and GISAXS, respectively. The dashed line corresponds to a linear fit with a slope of 0.995, and an intercept of 0.326 nm.

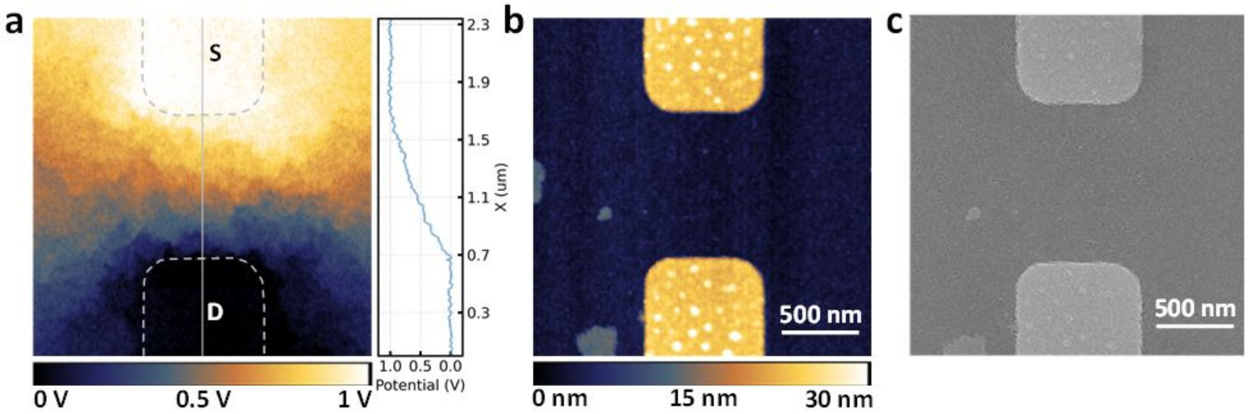

Figure S4. (a) Surface potential map and the corresponding line cut obtained from the FM-KFM scan of a working device based on GNP superlattice (OAm) without ligand exchange. A DC bias of 1 V was applied between source (S) and drain (D) electrodes. (b) The simultaneously obtained topography map. (c) SEM image of the same device.

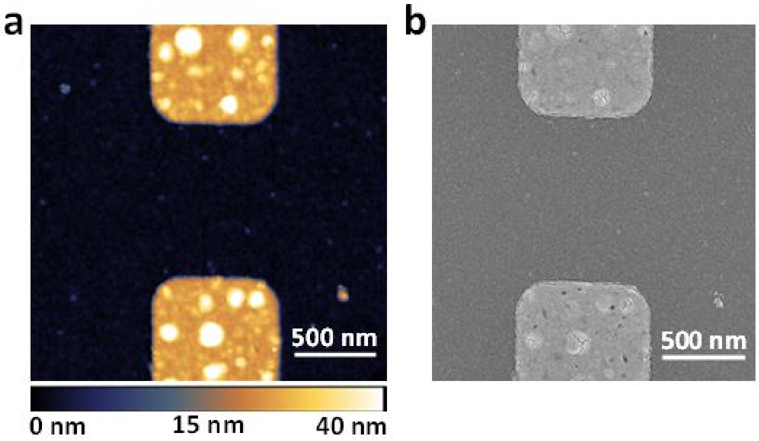

Figure S5. (a) The simultaneously obtained topography map from the same FM-KFM scan as Figure 3e. (b) SEM image of the same device.

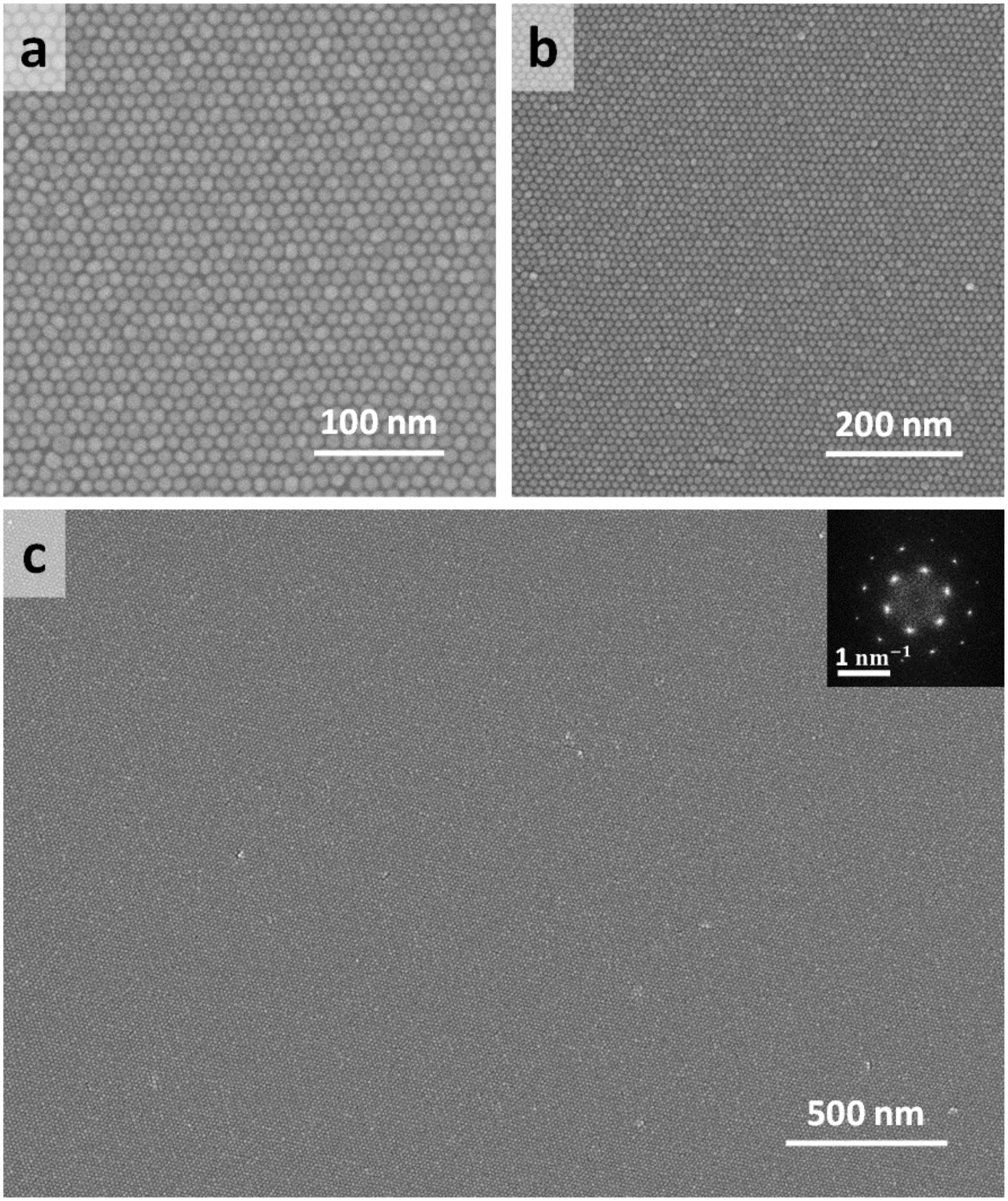

Figure S6. (a-c) SEM images of GNP superlattices after ligand exchange with $(NH_4)_2S$ (insert of (c), a 2D FFT power spectrum corresponding to a region of 2 by 2 μm^2 at the upper left corner of the SEM image).

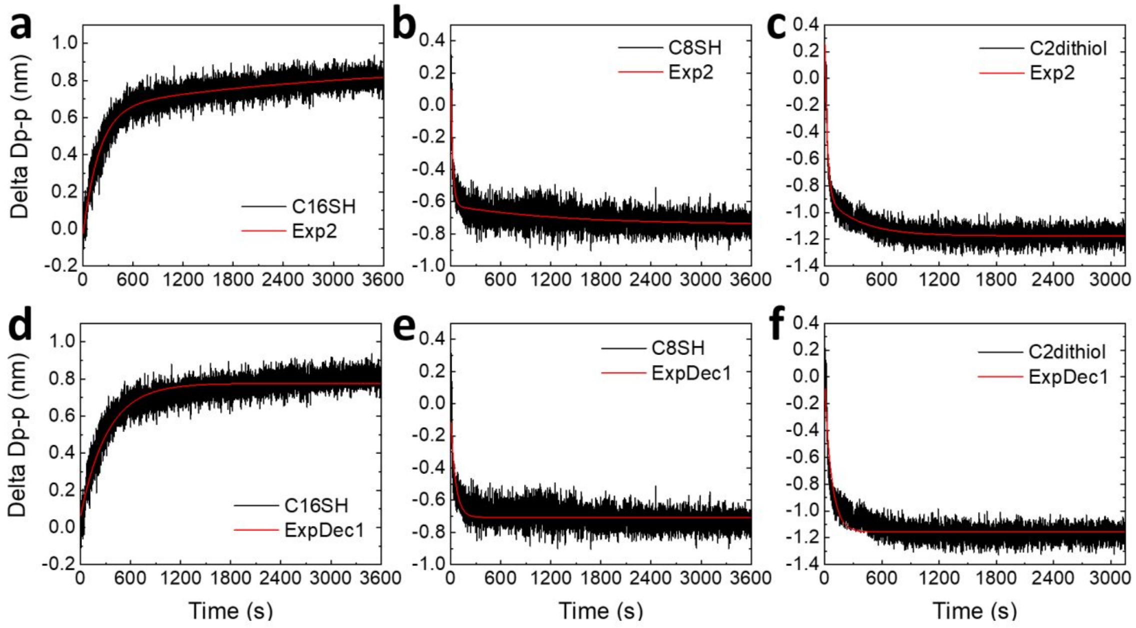

Figure S7. The *in situ* time evolution of the interparticle distance change during different ligand exchange processes, measured by GISAXS, fitted by a bi-exponential function (a-c), and an exponential function (d-f).

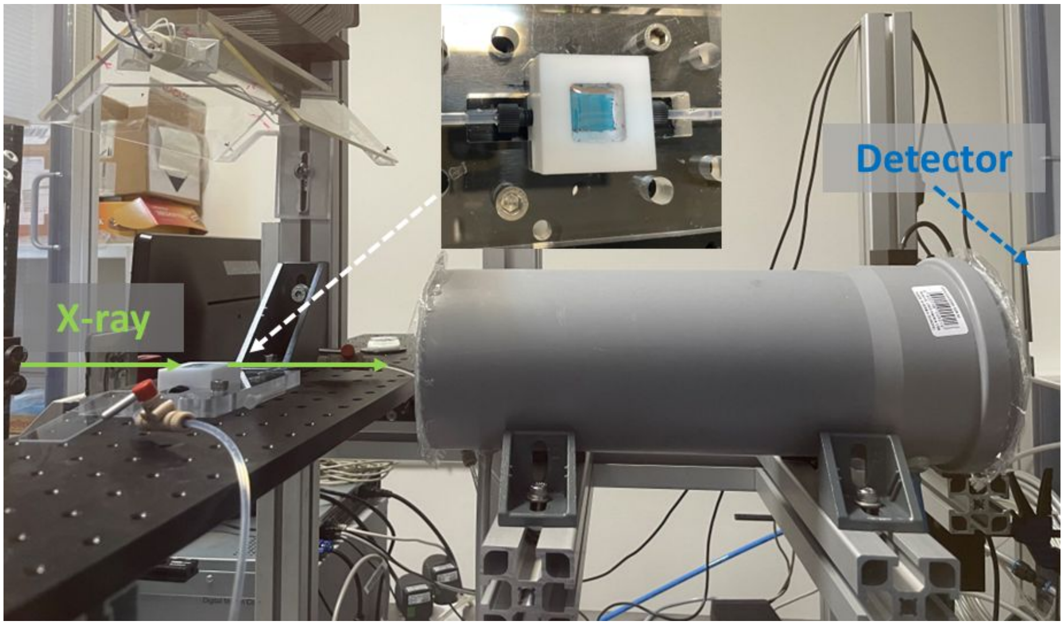

Figure S8. Photograph of the set-up for the *in-situ* GISAXS measurements.

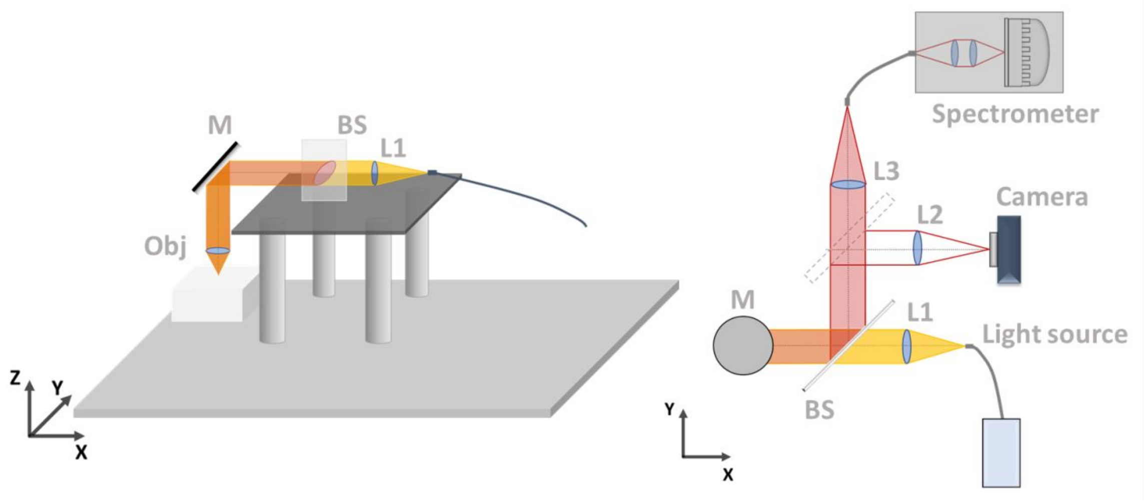

Figure S9. Schematic illustration of the optical set-up used for *in-situ* reflectance measurements during the ligand exchange process.

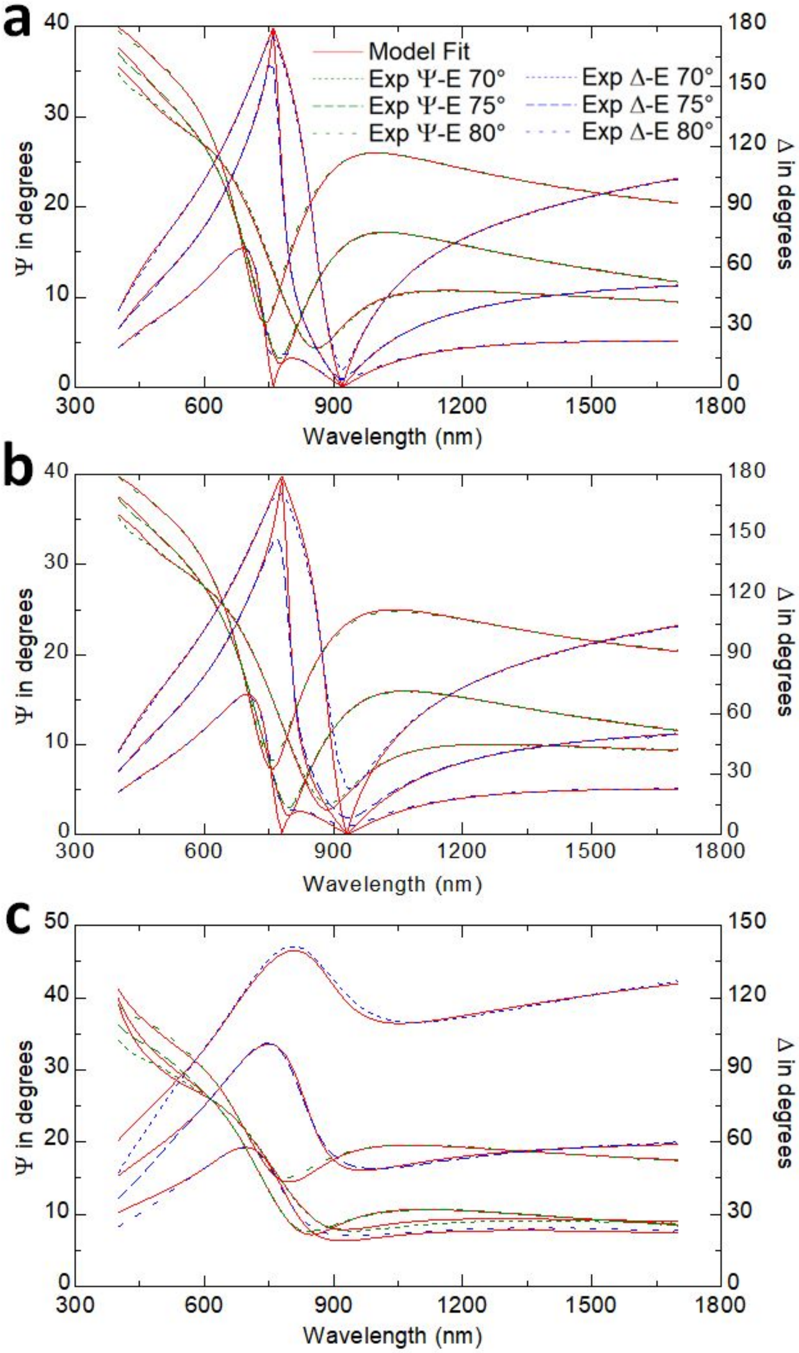

Figure S10. Amplitude (Psi) and phase (Delta) of the complex reflectance ratio from ellipsometry measurements at 70° , 75°, and 80° incidence angle. The samples correspond to GNP superlattices after ligand exchange with C2DT (a), 1,4-BDT (two-step) (b), and $(NH_4)_2S$ (c). 60 nm SiO_2/Si wafers were used as the substrate.

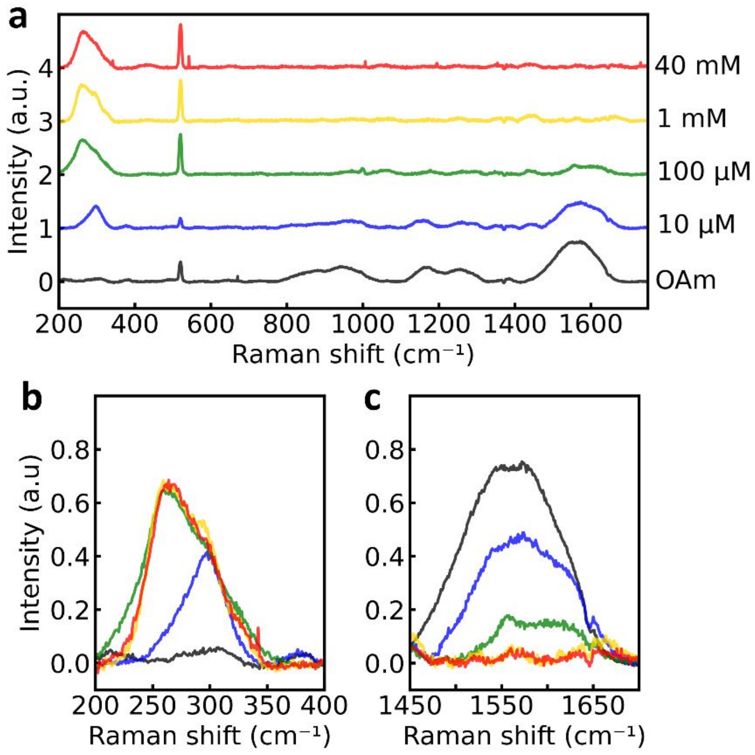

Figure S11. SERS spectra on GNP superlattices with OAm capping and after ligand exchange with $(NH_4)_2S$ of different concentrations. Backgrounds were subtracted using the common method of adaptive iteratively reweighted penalized least squares (arPLS).[4]

The Raman peaks at 1450-1700 cm^{-1} correspond to OAm ligands, including methyl, methylene, amine, and alkenyl stretching and bending vibrations.[5-8] The Raman peaks at 300 cm^{-1} and 265 cm^{-1} correspond to Au-S stretching vibration.[9] When 10 μM $(NH_4)_2S$ was used for ligand exchange, sulfide ligands first occupied the thermodynamically more favourable sites, resulting in the Raman peak at 300 cm^{-1}. With concentration of $(NH_4)_2S$ increased, sulfide ligands further occupied the thermodynamically less favourable sites, resulting in the Raman peak at 265 cm^{-1}. When 1 mM $(NH_4)_2S$ was used for ligand exchange, the vibration peaks from OAm ligands have already disappeared.

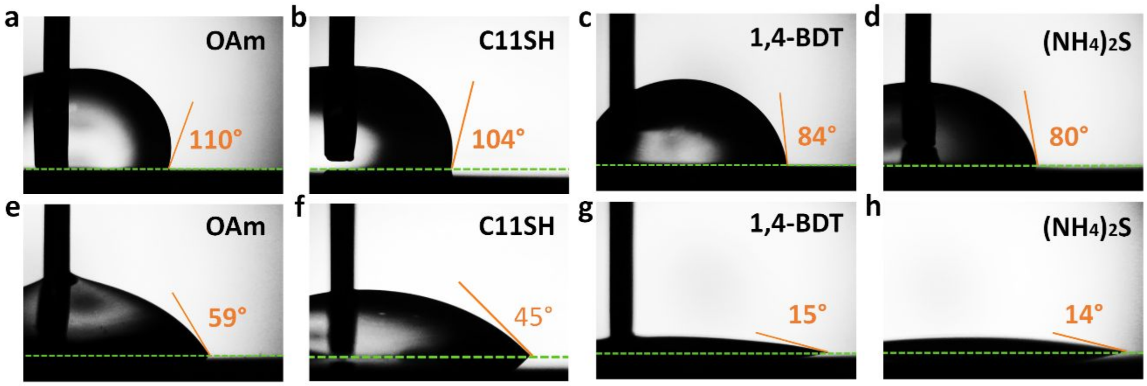

Figure S12. Images of a water droplet on top of GNP superlattices with OAm capping and after exchange with different ligands. The advancing contact angles are shown in (a-d) and receding contact angles are shown in (e-h). Significant change in the hydrophobicity of GNP top surfaces can be observed after ligand exchange.

References

(1) Read, D. T.; Dally, J. W. Theory of Electron Beam Moiré. *J. Res. Natl. Inst. Stand. Technol.* **1996**, *101*, 47–61. https://doi.org/10.6028/jres.101.007.

(2) Wen, T.; Majetich, S. A. Ultra-Large-Area Self-Assembled Monolayers of Nanoparticles. *ACS Nano* **2011**, *5*, 8868–8876. https://doi.org/10.1021/nn2037048.

(3) Eah, S. K. A Very Large Two-Dimensional Superlattice Domain of Monodisperse Gold Nanoparticles by Self-Assembly. *J. Mater. Chem.* **2011**, *21*, 16866–16868. https://doi.org/10.1039/c1jm11671a.

(4) Zhang, Z. M.; Chen, S.; Liang, Y. Z. Baseline Correction Using Adaptive Iteratively Reweighted Penalized Least Squares. *Analyst* **2010**, *135*, 1138–1146. https://doi.org/10.1039/b922045c.

(5) Wu, H.; Chen, W. Synthesis and Reaction Temperature-Tailored Self-Assembly of Copper Sulfide Nanoplates. *Nanoscale* **2011**, *3*, 5096–5102. https://doi.org/10.1039/c1nr10829h.

(6) Hou, X.; Zhang, X.; Yang, W.; Liu, Y.; Zhai, X. Synthesis of SERS Active Ag_2S Nanocrystals Using Oleylamine as Solvent, Reducing Agent and Stabilizer. *Mater. Res. Bull.* **2012**, *47*, 2579–2583. https://doi.org/10.1016/j.materresbull.2012.04.144.

(7) Perez De Berti, I. O.; Cagnoli, M. V.; Pecchi, G.; Alessandrini, J. L.; Stewart, S. J.; Bengoa, J. F.; Marchetti, S. G. Alternative Low-Cost Approach to the Synthesis of Magnetic Iron Oxide Nanoparticles by Thermal Decomposition of Organic Precursors. *Nanotechnology* **2013**, *24*. https://doi.org/10.1088/0957-4484/24/17/175601.

(8) Kluenker, M.; Mondeshki, M.; Nawaz Tahir, M.; Tremel, W. Monitoring Thiol-Ligand Exchange on Au Nanoparticle Surfaces. *Langmuir* **2018**, *34*, 1700–1710. https://doi.org/10.1021/acs.langmuir.7b04015.

(9) Parker, G. K.; Watling, K. M.; Hope, G. A.; Woods, R. A SERS Spectroelectrochemical Investigation of the Interaction of Sulfide Species with Gold Surfaces. *Colloids Surfaces A Physicochem. Eng. Asp.* **2008**, *318*, 151–159. https://doi.org/10.1016/j.colsurfa.2007.12.029.
